# Hemodynamic Alterations After Traumatic Amputation in the Immediate Postoperative Period

**DOI:** 10.7759/cureus.91616

**Published:** 2025-09-04

**Authors:** Lucas N Canaan, Morgan Krause, William Zickler, Bradley Kuhn

**Affiliations:** 1 Surgery, Northeast Georgia Medical Center Gainesville, Gainesville, USA; 2 Trauma and Acute Care Surgery, Northeast Georgia Medical Center Gainesville, Gainesville, USA; 3 Vascular Surgery, Northeast Georgia Medical Center Gainesville, Gainesville, USA

**Keywords:** general trauma surgery, lower limb amputation, lower limb trauma, orthopedic trauma surgery, trauma-related vascular injury

## Abstract

Background

Although long-term outcomes and chronic cardiovascular changes following amputation have been studied, there is limited literature on the acute hemodynamic alterations that occur in the immediate postoperative period of patients suffering from a traumatic amputation. Currently, no standardized management algorithms exist to guide care during this critical phase. This retrospective observational study aims to identify factors associated with cardiovascular changes and the relationship to morbidity and mortality in patients with traumatic amputations.

Objective

To evaluate acute cardiovascular effects and related clinical markers in patients undergoing traumatic amputations.

Methods

A retrospective observational study was conducted using data from the Northeast Georgia Health System Trauma Registry, a Level I community trauma center. Patients who sustained a traumatic amputation between January 2019 and September 2024 were included.

Results

A total of 34 patients met the inclusion criteria. Although the small sample size limited statistical power, several notable trends were observed. Patients who underwent above-knee amputation (AKA) were more likely to require continuous renal replacement therapy (CRRT) and received higher volumes of blood products compared to those with below-knee amputations (BKA). Additionally, patients who died had higher injury severity score (ISS) than survivors.

Conclusion

While no definitive clinical recommendations could be established due to the limited sample size, the findings highlight key trends that merit further investigation. Future studies at high-volume centers or through multicenter collaborations may better elucidate the acute cardiovascular impacts of traumatic amputation and support the development of targeted management strategies.

## Introduction

Traumatic injuries present significant challenges to patient outcomes, with prognosis largely depending on the severity and nature of the injury. One of the most devastating types of trauma is traumatic amputations, which carry substantial physiological and psychological burdens. While many patients survive the initial hospitalization and proceed to recovery, a notable subset experience severe complications, with some progressing to early mortality. This retrospective observational study examines a cohort of patients who died shortly after admission following either a traumatic amputation sustained in the field or an emergent amputation performed soon after arrival at a trauma and acute care surgery service.

The long-term consequences of limb amputation have been well documented. Regardless of concurrent traumatic injuries, limb amputations are associated with complications that affect mobility, quality of life, and overall survival. Although direct comparisons between traumatic and non-traumatic (e.g., vascular-related) amputations are complicated by differing etiologies, there is some overlap in outcomes. For instance, a retrospective chart review by Chopra et al. found that fewer than 50% of patients returned to ambulation after a major lower extremity amputation [1]. Functional decline was particularly pronounced in patients with comorbid conditions such as obesity, frailty, dementia, and renal failure requiring dialysis. 

Despite a growing body of literature focused on long-term recovery and rehabilitation, there is a notable gap in research concerning the acute cardiovascular and hemodynamic changes that occur perioperatively in patients undergoing traumatic amputations. The early phase of critical illness, particularly immediately after severe injury, is a vulnerable period during which subtle but significant changes in cardiac physiology may occur. Cardiac arrhythmias, for example, are not uncommon following major trauma. In a recent study of 1,119 acutely traumatized patients, 326 (29.1%) experienced arrhythmias, a complication associated with longer ICU and hospital stays and increased in-hospital mortality [2]. 

The objective of this study is to analyze the early clinical course and cardiovascular responses in patients with traumatic amputations admitted to a community-based Level I trauma center, with particular attention to those who expired shortly after presentation. These findings aim to inform future research and support the development of evidence-based protocols for acute management in this high-risk population.

## Materials and methods

Study design 

This retrospective observational study was conducted at Northeast Georgia Medical Center, a community Level 1 trauma center between January 2019 and September 2024. The focus was patients who have had traumatic amputations with all amputations being carried out by either the vascular surgery team or orthopedic surgery team.

Population

This study population inclusion criteria are patients greater than 18 years old and less than 89 years old. The amputation was done by either vascular surgery or orthopedic surgery exclusively as trauma surgery does not typically perform amputations at this facility. The amputation inciting event was secondary to a traumatic insult and not due to another event such as an embolic phenomena or peripheral arterial disease. Finally, the patient's are managed exclusively by the trauma and acute care surgery service as primary team.

The exclusion criteria include amputations secondary to nontraumatic pathology. Additionally, patient's less than 18 years but greater than 89 years were excluded from the data set. Patients with incomplete data were also excluded.

Data sources

The data source utilized for this study was the Trauma Registry. The data included admission and discharge dates, patient demographics (age, race, sex), surgery data, surgery notes, length of stay, past medical history, encounter data, hospital costs, complications, and operative times. Additional data collected from the patients included operative pictures or videos, which were de-identified prior to use. The primary investigator maintained control of the data and assigned a number to each operation's media to allow for its future use in a de-identified manner.

Ethical considerations

This is a retrospective study and individual consent is not required. IRB approval completed prior to study and found to be exempt.

Statistical analysis

Given small sample size, statistical analysis was unable to be completed. All results will be observational in nature.

## Results

**Figure 1:**
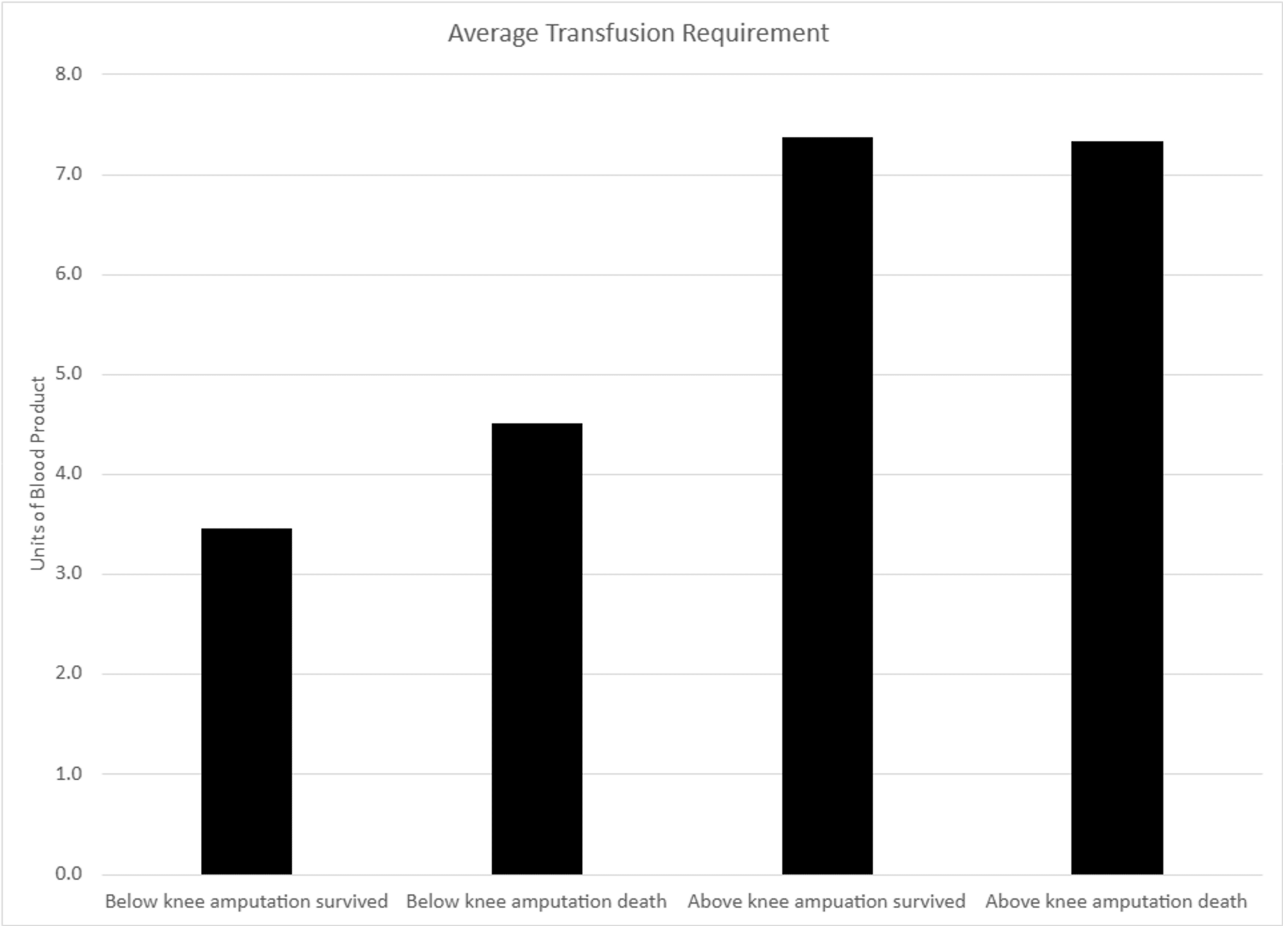
Average transfusion requirements versus total units of all blood products given during hospital course.

**Figure 2:**
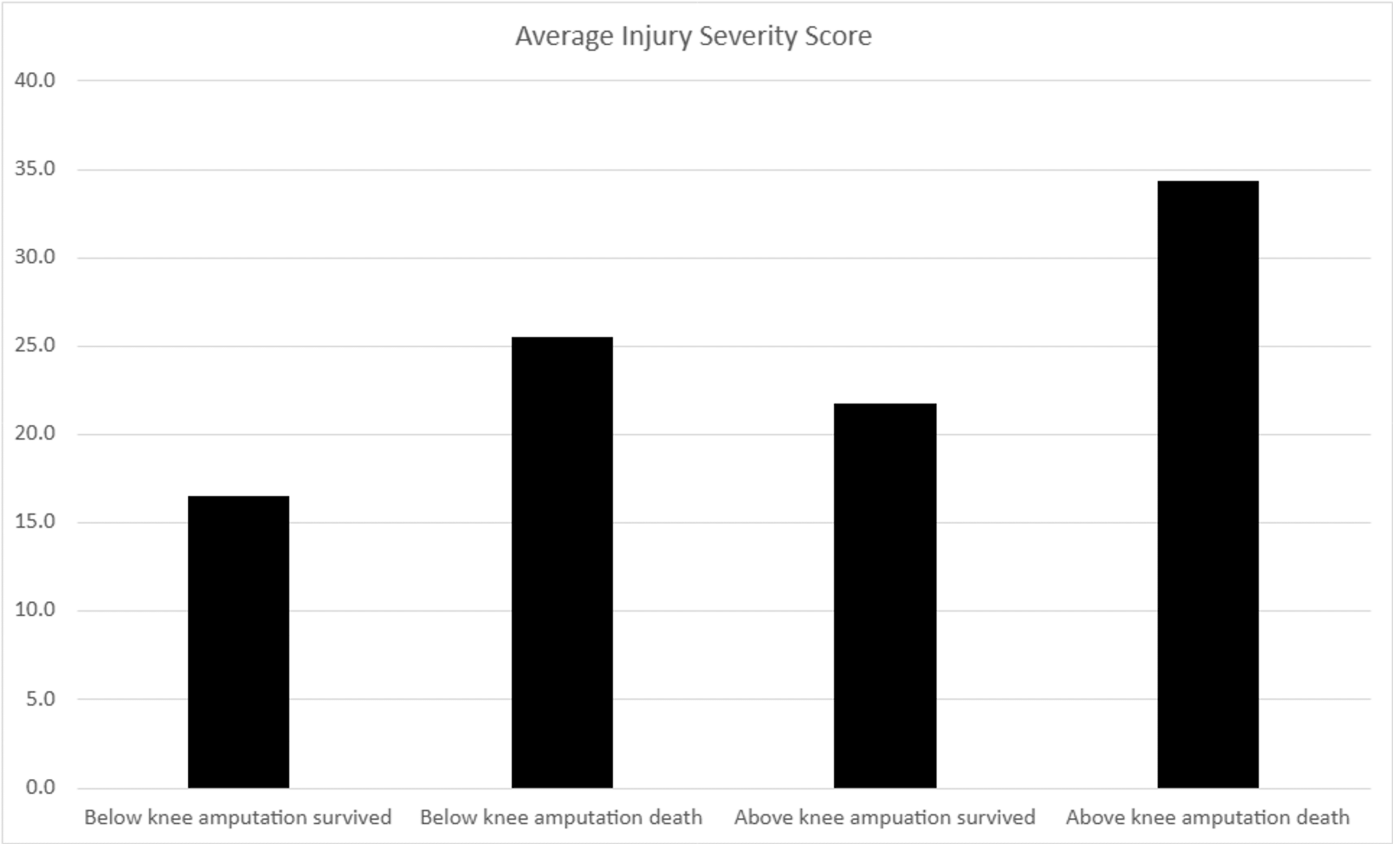
Average ISS as compared to the amputation type. ISS: injury severity score.

Evaluation of the data was challenging due to the small size of the dataset and differences in management among the patients. The complete patient dataset can be seen in Table 1. The patients were predominantly male (28 males versus 6 females). There were more below-knee amputations (BKA) than above-knee amputations (AKA). Vascular reconstruction was attempted in five of the 34 patients (14.7%) with all ultimately needing an amputation. Additionally, five of the 34 patients (14.7%) required continuous renal replacement therapy (CRRT). Among these five patients, one patient (20%) was within the surviving BKA group, while the other four patients (80%) were within the AKA group (two survived and two expired). The overall transfusion requirement was higher in the AKA group, regardless of survival status, compared to the BKA group. This comparison is shown in Figure 1. The average injury severity score (ISS) was highest in the AKA group that expired, followed by the BKA group that also expired. This comparison is demonstrated in Figure 2.

**Table 1:** Patient demographics and breakdown of amputation patterns. AKA: above-knee amputation; BKA: below-knee amputation; CRRT: continuous renal replacement therapy.

Patient demographics	
Male	28
Female	6
Average patient age	
Male	46 years
Female	59 years
Vascular reconstruction attempted	
BKA survived	1
BKA death	1
AKA survived	2
AKA death	1
Amputation location	
BKA	22
AKA	12
Survived	
BKA	20
AKA	2
Expired	
BKA	9
AKA	3
Required CRRT	
BKA survived	1
BKA death	0
AKA survived	2
AKA death	2

**Figure 3:**
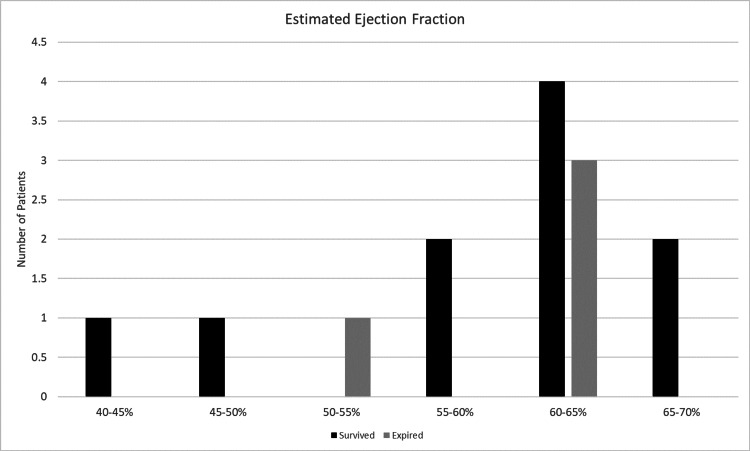
Comparison of ejection fraction between patients who survived and those who expired.

Of the patients who expired, one had a secondary severe closed head injury, one died 31 days after admission due to cardiopulmonary arrest secondary to sepsis, one patient died from multiorgan failure largely due to hepatorenal syndrome, and the final two died secondary to cardiopulmonary arrest within days of admission. Of the three patients passed away near their time of admission, the patient's echocardiogram did not show any severe structural abnormalities or decreased ejection fraction. No direct correlation could be made with patients who survived, as not all patients received an echocardiogram as part of their workup. The available data do not show any difference among patients who survived or expired. Full data can be seen in Figure 3.

## Discussion

Key findings

The primary aim of this study was to evaluate cardiovascular changes in patients who experienced traumatic amputations. However, due to the limited sample size, the study was underpowered to allow for robust statistical analysis or definitive conclusions. As a result, the current study design does not fully address the original research question. Despite this limitation, the findings provide preliminary insights and underscore the need for future studies with larger cohorts. In the long term, it may be valuable to develop a predictive algorithm for perioperative cardiovascular changes based on the type and level of amputation. Such a tool could optimize patient care and enhance the allocation of critical resources.

One of the key observations from this study was that patients who died had higher ISS than those who survived. This finding is consistent with established literature demonstrating that ISS is linearly associated with morbidity, mortality, and hospital length of stay [3]. Another area of interest was left ventricular ejection fraction, which showed no meaningful difference between survivors and non-survivors, suggesting that baseline cardiac function may not have been a major differentiating factor in this cohort.

Blood product utilization revealed additional insights. Patients in the AKA group received more blood products overall compared to those in the BKA group, regardless of outcome. However, among patients within the AKA group, there was no difference in transfusion volume between survivors and non-survivors. While the findings do not establish causation, they align with prior work by Robinson et al., who identified blood transfusion volume as an independent predictor of mortality in patients with intra-abdominal hemorrhage [4]. Though their study did not focus on traumatic amputations, the association between increased transfusion requirements and injury severity appears consistent.

Strengths

Strengths of this study include the detailed presence of data. This allowed for detailed examination and evaluation of the patients within this trauma population.

Limitations

The limitations of the study are largely related to the sample size. Due to the small sample size, statistical analysis could not be completed. Additionally, given the multifactorial components of acute traumatic injuries, there is room for confounding variables leading to patient's dying. As was seen in one patient, while there might have been a component of deterioration secondary to amputation, a severe closed head injury can lead to patient death. To better appreciate and isolate the cardiovascular changes that occur in the acute perioperative setting following amputation, a larger sample size would be needed. Other limitations would be the diverse severity associated with each patient. For example, patients with an isolated limb injury will receive a focused workup, whereas those in a high-energy motor vehicle collision will receive a more extensive workup to detect other injuries.

Implications

While definitive recommendations cannot be made based on this data set, there are interesting questions that have developed. In both the BKA and AKA groups, patients who died had a higher ISS compared to those who survived. This finding aligns with the classifications described by Javali et al., where an ISS greater than 15 indicates moderate trauma, 16-25 denotes severe trauma, and scores above 25 reflect profound trauma. These results underscore the importance of closely monitoring this high-risk patient population, as they are more likely to experience clinical deterioration.

Patients who underwent an AKA were more likely to require CRRT compared to those who received a BKA. Both procedures reduce the overall fixed vascular volume; however, it can be inferred that an AKA may lead to more pronounced hemodynamic alterations due to the greater loss of the vascular compartment relative to a BKA. This increased physiological impact may affect circulating volume and contribute to cardiovascular instability. As such, these findings suggest that patients undergoing AKA may benefit from closer monitoring of cardiac function, with particular attention to fluid balance and early identification of either hypervolemia or hypovolemia, depending on the course of initial resuscitative efforts.

Patients with a BKA who died received a higher average volume of transfused blood compared to survivors. Furthermore, patients with an AKA received more blood products overall, regardless of survival status. Although blood transfusion volume is not a direct measure of injury severity, it may serve as a surrogate marker, as increased transfusion requirements often correlate with more severe trauma. Substantial blood product administration also alters intravascular volume, and in the setting of acute cardiovascular changes, particularly due to the loss of fixed vascular space following amputation, this may impact overall hemodynamic stability. In this context, careful fluid management and close cardiac monitoring, including the use of routine echocardiography or other noninvasive cardiac assessment tools, may be beneficial in optimizing outcomes for this high-risk patient population.

## Conclusions

While definitive conclusions or clinical recommendations cannot be drawn from this study due to its limited sample size, several notable findings emerged. Overall, the results were consistent with existing literature and contribute to the growing body of knowledge surrounding traumatic amputations. The primary question regarding acute cardiovascular changes in patients undergoing traumatic amputations warrants further investigation, ideally through higher-volume institutional studies or multicenter collaborations. Such research could ultimately lead to improved patient outcomes through the development of standardized management protocols.
